# Effects of Social Group Housing on the Behavioral and Physiological Responses of Captive Sub-Adult Giant Pandas

**DOI:** 10.3390/ani14172545

**Published:** 2024-09-02

**Authors:** Bo Yuan, Qin Fu, Xue-Ying Wang, Xiao-Hui Zhang, Yu-Liang Liu, Rong Hou, Ming-Yue Zhang

**Affiliations:** 1 Chengdu Research Base of Giant Panda Breeding, Chengdu 610081, China; boom@panda.org.cn (B.Y.); fuqin0416@163.com (Q.F.); wangxuey_91@163.com (X.-Y.W.); xiaohuizhang1986@163.com (X.-H.Z.); liuyuliang@panda.org.cn (Y.-L.L.); hourong2000@panda.org.cn (R.H.); 2 Sichuan Key Laboratory of Conservation Biology for Endangered Wildlife, Chengdu 610081, China; 3 Sichuan Academy of Giant Panda, Chengdu 610081, China

**Keywords:** sub-adult captive giant panda, playing behavior, stereotyped behavior, fecal microbes, cortisol, heart rate variability

## Abstract

**Simple Summary:**

Giant pandas (*Ailuropoda melanoleuca*) are a critically endangered relict species, often referred to as a “national treasure” and a “living fossil”. China has implemented an ex situ conservation model in order to safeguard and revitalize the population. Given their highly solitary nature, sub-adult giant pandas typically undergo a phase of group living during the ex situ protection process aimed at optimizing space and management efficiency. However, as a solitary species, giant pandas’ behavioral responses and physiological performance in social settings remain elusive. This study aims to address these questions by observing the daily behavior of giant pandas through photography and analyzing their cortisol levels and intestinal microbial identification to gain insights into their physiological performance. Based on our current findings, we have determined that the group-rearing model during the sub-adult stage benefits the overall welfare of adult captive giant pandas within their current captive environment.

**Abstract:**

Wild giant pandas are inherently solitary creatures, however, the ex-situ conservation efforts significantly alter the living circumstances of their captive counterparts. Following the breeding period, giant pandas in captivity may be maintained in social groups. Currently, there is a lack of research on the effects of group housing on the physiology, behavior, and gut microbiota of captive giant pandas. This study divided six captive giant pandas into two groups following the breeding period. By comparing the behavior, physiology, and microorganisms of the two groups, we aim to investigate the behavioral responses and physiological adaptation mechanisms exhibited by captive giant pandas in a “group living” state. Our findings indicate that sub-adult giant pandas housed in group settings exhibit a significantly longer duration of playing behavior (including interactive and non-interactive play) compared to their counterparts housed separately (*p* < 0.001) while also demonstrating a significantly lower duration of stereotyped behavior than their separately housed counterparts. Additionally, an analysis of urine cortisol and heart rate variability between the two groups revealed no significant differences. Simultaneously, the group housing strategy markedly elevated the β diversity of gut microbiota in sub-adult giant pandas. In conclusion, the group-rearing model during the sub-adult stage has been shown to significantly alter the behavioral patterns of captive giant pandas. In conclusion, within the present captive setting, the group-rearing approach during the sub-adult stage proved to be less distressing for adult captive giant pandas.

## 1. Introduction

The giant panda (*Ailuropoda melanoleuca*) is among the most critically and is classified as an endangered species on the IUCN Red List [1]. Due to habitat destruction, fragmentation, human interference, and other factors, the wild giant panda faces the threat of population isolation, reduction in genetic diversity and potential extinction [2]. Since the 1980s, the Chinese government has established several large panda relocation protection and breeding institutions, such as the Giant Panda Conservation and Research Center and the Chengdu Research Base of Giant Panda Breeding. These institutions are dedicated to the rescue, breeding, wild release, and scientific research of giant pandas in order to protect this rare species [3]. Through a series of scientifically rigorous and comprehensive strategies, researchers have successfully addressed the challenges associated with estrus difficulty, fertilization difficulty, and young survival in the captive breeding of giant pandas. As of 2021, the global population of captive giant pandas has reached 673, achieving self-sustainability and high-quality development within captive populations while also contributing to the support of wild giant panda populations [4]. After rigorous training, artificially bred giant panda individuals can be reintroduced into small wild populations facing the threat of extinction, thereby effectively bolstering the population and contributing to the breeding efforts within these vulnerable wild populations. This approach represents a crucial strategy for safeguarding small populations in their natural habitat. Through years of dedicated efforts, 10 captive giant pandas have been successfully released into the wild in China, facilitating the recovery of the small population of giant pandas in Xiaoxiangling and at the southern end of the Minshan Mountain range [5]. The findings from the fourth 10-year survey of the giant panda population, conducted by the National Forestry and Grassland Administration of China, indicate a notable increase in the number of wild giant pandas to 1864 compared to the previous survey [6]. Consequently, there has been a shift in the conservation status of giant pandas from “endangered” to “vulnerable” [7].

In comparison to the natural habitat of wild giant pandas, the living conditions of giant pandas have undergone significant changes during ex-situ conservation, particularly during the nursery and sub-adult stages. These changes include premature weaning, separation from mothers, non-parental rearing and care for twins, as well as group management of multiple giant pandas during their sub-adult stage [8]. These environmental differences result in variations in behavioral patterns between wild and captive giant pandas, including the manifestation of stereotyped behavior frequently observed in captive individuals diverging from their natural habitat [9]. The prevailing consensus suggests that the transition of wild animals from their natural habitat to a captive environment may constrain the typical expression of certain instinctive behaviors, resulting in unmet instinctual needs and ultimately leading to a deterioration in animal welfare [9,10]. We observed that captive giant pandas exhibit a suite of stereotyped behaviors, including circling, pacing, and head shaking [11,12], a phenomenon that has not been documented in their wild counterparts. Stereotyped behavior is widely believed to reflect a suboptimal environment and inadequate animal welfare conditions [13,14].

Wild giant pandas are typically solitary animals. With the exception of the brief estrus season in spring each year, during this time, they communicate their reproductive status through chemical and acoustic signals, ultimately attracting mates and coordinating mating activities [15,16]. During non-estrus seasons, wild pandas have minimal social interaction [17]. When giant pandas in the wild give birth to twins, the demanding natural environment and the vulnerability of newborns often lead to the maternal abandonment of one cub, resulting in predominantly solitary rearing for most offspring [18]. At 18–24 months old, the cubs are weaned from their mothers and begin independent living [17]. This indicates that wild giant pandas seldom form groups with peers during the entire sub-adult period. Research has revealed that giant pandas exhibit limited territorial behavior during the sub-adult stage and can coexist harmoniously with their companions. Consequently, in order to optimize breeding space and streamline management, captive institutions often house 5–7 sub-adult giant pandas together until they reach adulthood [9]. Bears are generally solitary, with the exception of mothers with their young [19]. Their preference for solitude is primarily driven by the need to efficiently locate food, minimize competition, and conserve energy. However, in captive environments with a stable food supply, absence of predators, and limited space, bears may display social behavior, forming “captive social management” groups. This stereotypical behavior exhibited by captive bears is attributed to their inability to fulfill their natural needs [20,21,22]. When giant pandas change from harsh environments in the wild to captive environments, they no longer face harsh weather and lack of food, etc. We have been thinking about whether they can accept social life after the end of their breeding period and how their social interaction has changed. Currently, there is limited research on the impact of captive management patterns (human intervention during breeding and early adulthood, as well as group living environment during sub-adulthood) on the behavior of giant pandas. Our previous study revealed that the confinement, restricted space, and interference from various non-natural stressors (such as tourist noise, changes in enclosures, and unfamiliar odors) in artificial captive-rearing environments could impose stress on female giant pandas during their maternal period. However, the levels of behavioral diversity exhibited by young giant pandas raised in different rearing environments vary, with significantly higher levels observed in those raised through natural nursing compared to those under artificially assisted parenting [8]. Following the reproductive phase, giant pandas may experience social group rearing. However, there is no research on their behavior within this group setting.

Hence, the primary objective of this study is to investigate the behavioral response and physiological changes of giant pandas, which are typically solitary animals but may be kept in groups in captivity. The focus of this study is to investigate the social effects of captivity on the behavioral development and expression of giant pandas that are accustomed to solitary living. Additionally, we will measure levels of urine cortisol, degree of HRV, and fecal microbiota. Ultimately, our research aims to explore the impact of captive social management practices on sub-adult giant pandas during ex-situ conservation efforts.

## 2. Materials and Methods

### 2.1. Animals and Feeding Management

In this study, we selected six sub-adult captive giant pandas from the Chengdu Research Base of Giant Panda Breeding (Panda base) as the research subjects. The giant pandas were divided into two groups: a solitarily managed individual group (SMI) and a socially managed group (SMG) after weaning, each consisting of 3 giant pandas (Table 1). The family relationship of each giant panda is also shown in Table 1. The SMG group was raised together after weaning, while the SMI group was raised separately after weaning, which typically occurs between 1.5 and 2 years old. Each group of giant pandas consists of two males and one female. Prior to data collection, the experimental giant pandas were housed either in group settings or individually for a duration exceeding 7 months. During their sub-adult period, both the social group and the solitary group of giant pandas resided in an indoor enclosure connected to an outdoor playground. Two cohorts of experimental giant pandas were raised in two similar indoor semi-natural breeding environments during the daytime and housed in unified indoor enclosures (3.71 m × 7.12 m) at night or during hot weather conditions. The indoor semi-natural environments cover an area exceeding 152 square meters, each equipped with animal drinking devices and air conditioners. Three pandas are housed individually in three separate semi-natural environments and fences, while the remaining three pandas cohabitate in one shared environment and fence. Giant pandas were released into the outdoor playgrounds at 8:30 each day and brought indoors at 17:30. Feeding occurred at 8:00 and 13:00 daily with an ample bamboo supply available. Additionally, intermittent feeding of “Wowotou”, apples, honey water, and medicine took place during intervals. In all indoor animal enclosures, a single water source was provided, with additional ad libitum access to water.

### 2.2. Behavior Observation and Data Collection

We conducted filming of the giant panda from late July to September 2023, utilizing a Sony 4K camcorder. The temperature of the outdoor playgrounds was maintained at 28.5 ± 6.2 °C with a relative humidity of 78 ± 2% from 24 July 2023 to 18 September 2023. Each giant panda was filmed for a duration of 6 days, with daily filming sessions lasting 4 h (Each panda was filmed for a total of 24 h), commencing at 9:00 AM and concluding at 11:00 AM, and then resuming from 14:00 PM to 16:00 PM, respectively, avoiding the half hour that the breeder cleaned manure, which occurred from 13:00 h to 13:30 h, see Table 1 for specific groups. The video footage captured the feeding behavior, resting behavior, playing behavior and stereotyped behavior of each giant panda. Table 2 shows the definitions of these behaviors [8,23,24,25]. A continuous recording method [24] was employed to systematically record the behaviors throughout the filming period, with durations of feeding, resting, playing and stereotyping behaviors being meticulously documented.

### 2.3. Determination of Heart Rate Variability (HRV)

Heart rate variability (HRV) refers to the fluctuation in heart rate or the duration of the R-R interval (heart cycle) and has emerged as a crucial tool for assessing risk [26]. The standard deviation of average normal-to-normal intervals (SADNN) denotes the mean standard deviation of R-R intervals, measured in milliseconds, and serves as an indicator of HRV. Electrocardiograms (ECG) were continuously monitored without anesthesia for three consecutive days in each experimental giant panda. During the period from October to December 2023, behavior training was conducted for giant pandas in order to facilitate the measurement of their heart rate variability. Subsequent real-time monitoring took place between January and February 2024, specifically from 9:00 to 10:00 each morning. The duration of monitoring sessions ranged from 2 to 5 min, depending on the condition of the giant panda. In our study, we employed an innovative smart wearable heart monitoring system capable of accurately measuring animal ECG and exporting heart rate R-R interval data through specialized software developed by Jiangsu Elansen. Subsequently, the SDANN values for two groups of giant pandas were tabulated and calculated. We conducted heart rate monitoring on two distinct cohorts of giant pandas and subsequently performed rigorous measurements and calculations to determine their HRV.

### 2.4. Measurement of Urinary Cortisol

Due to the limited sample size, we employed a method of collecting urine 3–4 times per month for each giant panda as the repeated sample size, with urine collection consistently taking place between 7:00 AM and 8:00 AM. We collected a total of 116 urine samples from six giant pandas between January to mid-July 2023. Each giant panda collects at least three urine samples every month. The concentration of cortisol in the urine was measured using an ELISA kit (Cayman, Ann Arbor, MI, USA). After the panda has voided, it should be gently guided into a nearby enclosure and the urine should be collected in a 2 mL frozen storage tube using a sterile syringe. Careful attention should be paid to selecting uncontaminated and undiluted urine, with clear labeling of the date, time, and individual identification. The urine sample should then be promptly sealed with a film without any chemical treatment, refrigerated, transported back to the laboratory, and stored in a −80 °C freezer for analysis. The concentration of cortisol in urine was measured using the Cayman Cortisol ELISA Kit (No. 500360) with a sensitivity of 35 pg·mL^−1^, an intra-assay coefficient of variation less than 13.4%, and an inter-assay coefficient of variation less than 25.8%. The standard procedure involved preparing the standard curve and diluting the samples before adding ELISA buffer, standard solution, sample aliquot, cortisol tracer reagent, and cortisol antibody to the microplate coated with goat anti-mouse immunoglobulin. The plate was then incubated at 4 °C overnight. Subsequently, after washing the plates on the following day, Ellman’s reagent and tracer were added, followed by incubation in darkness for 1.5~2 h before measuring absorbance at 405 nm using a Thermo Scientific Multiskan MK3 spectrophotometer (Thermo Fisher Scientific, Waltham, MA, USA).

Creatinine (Cr) assays were conducted by diluting the urine 20 times and adding it to a 96-well plate with 0.05 mL of 0.04 mol × L^−1^ picric acid and 0.05 mL of 0.75 mol × L^−1^ NaOH. After incubation at room temperature (25 °C) for 15 min, the optical density (OD) value at 492 nm was measured using a Thermo Scientific Multiskan MK3 spectrophotometer. Samples with a creatinine value lower than 0.1 mg × mL^−1^ were excluded from further analysis due to potential water contamination based on the OD value not being used in these cases. Creatinine values were utilized to standardize for variations in water content within each urine sample, where the hormone concentration in each urine sample was divided by the creatinine concentration using specific formulas: A = creatinine content (mg × mL^−1^) = [creatinine (μg × mL^−1^) × dilution ratio]/1000; B = cortisol content (ng × mL^−1^) = cortisol content per well (ng/well) × dilution release ratio; C = creatinine to correct cortisol (cortisol ng/mg Cr) = B/A.

### 2.5. 16S rDNA Amplicon Sequencing

We conducted a study on the microorganisms present in the feces of giant pandas, with 15 samples in each group and a total of 30 fresh samples collected within 10 min after excretion. During the period of behavioral filming from late July to September 2023, we collected fecal samples. The samples were promptly stored at −80 °C for testing following collection. Our research methodology was based on the approach outlined by Zhang et al. (2023) for microbial determination [27]. Genomic DNA from the fecal microorganisms was extracted using Omega’s Mag-bind soil DNA kit and subsequently assessed for purity and concentration through 1% agar-gel electrophoresis. Following this, appropriate samples were selected for amplification in a centrifuge tube. The TruSeq ^®^ DNA PCR-Free Sample Preparation Kit was utilized to construct the library, and Illumina NovaSeq 6000 sequencer PE250 read-length computer sequencing was employed. Data from each sample were distinguished based on barcode sequences, with reading data extracted from both ends of sequencing. After quality control filtering to assess read quality and merge effectiveness, we obtained valid data, which were then analyzed accordingly. The sequencing process was carried out by Shanghai Applied Protein Technology Co., Ltd. (Shanghai, China).

The α and β diversity indices were calculated using Qiime2 software (version number: 2020.8). α diversity was determined by rarifying the OTU table and calculating three metrics: (1) the Chao1 index, (2) the ACE index, and (3) the Simpson index. Rarefaction curves were generated based on these metrics. QIIME computed both weighted and unweighted UniFrac distances, which are phylogenetic measures of beta diversity. The unweighted UniFrac distance was utilized for principal coordinate analysis (PCoA), employing the unweighted pair-group method with arithmetic mean (UPGMA) for clustering. The principal coordinates obtained from PCoA facilitated the visualization of complex, multi-dimensional data coordinates. The LEfSe method was employed to quantitatively analyze biomarkers across distinct groups. Utilizing the inter-group difference test approach, based on the community abundance data acquired, a rigorous statistical method was utilized to conduct hypothesis testing for microbial community species between two or multiple groups of samples, evaluating the significance level of species abundance differences and obtaining information on species with significant differences between the groups.

### 2.6. Data Statistics and Analysis

The behavioral test data were analyzed using the Statistical Package for Social Science (SPSS 22.0; IBM Institute Inc., Chicago, IL, USA). Due to the small sample size, a Shapiro–Wilk test was conducted to assess normal distribution, and the Explore function in Descriptive Statistics was utilized to examine variance homogeneity. Subsequent analysis revealed that all behavioral and HRV data met the criteria for normal distribution and variance homogeneity. A total of 19 values that were deemed too large or too small were removed using SPSS software (version number: 22.0), resulting in 97 valid data points that satisfied normal distribution. Subsequently, an independent sample *t*-test was conducted to assess the significance of the difference, with all results reported as mean and standard error (Mean and SEM). A *p*-value > 0.05 was considered insignificant between the two groups, while a *p*-value < 0.05 was deemed significant. Significant differences between different groups were denoted by * (* *p* < 0.05, ** *p* < 0.01, ***p < 0.001).

## 3. Result

### 3.1. Behavioral Differences of Giant Pandas under Different Feeding Modes

**Figure 1:**
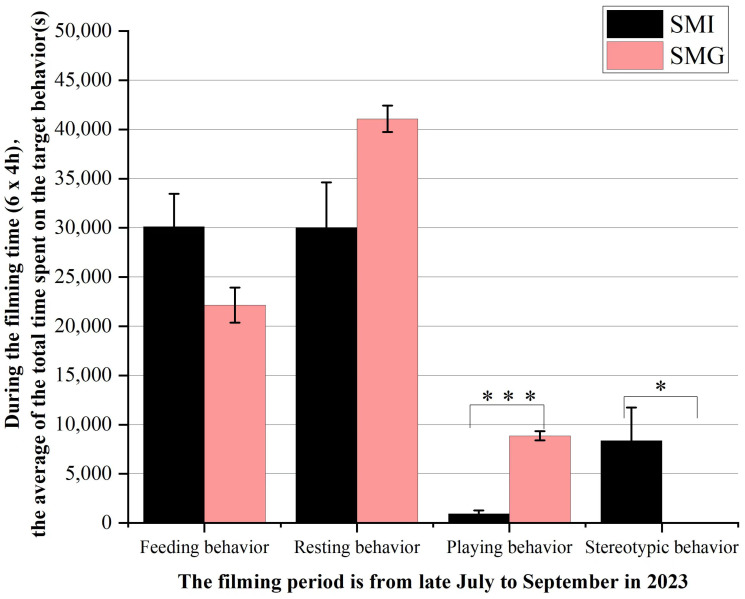
**Behavior statistics of two groups of giant pandas.** The abscissa represents feeding behavior, resting behavior, playing behavior and rigid behavior. The ordinate indicates the total time spent on the feeding behavior, resting behavior, playing behavior and stereotyped behaviors during the observation period, measured in seconds (s). * *p* < 0.05; *** *p* < 0.001. n = 3 per group.

Upon analyzing the behavior of giant pandas under various feeding modes, the findings are presented in Figure 1. There was no statistically significant difference in the duration of feeding and resting behaviors between the SMI and the SMG group (*p* > 0.05). Notably, giant pandas in the SMG group exhibited a significantly higher amount of time engaged in playing behavior compared to those in the SMI group (*p* < 0.001) while also demonstrating a significantly lower duration of stereotyped behavior than their SMI counterparts (*p* < 0.05).

### 3.2. Difference of Heart Rate Variability under Different Feeding Modes

The results of the HRV analysis are presented in Table 3. The SDANN value for the SMI group was determined to be 56.541 ± 23 ms, while that for the SMG group was found to be 29.505 ± 12 ms. Statistical analysis revealed no significant difference between the two groups.

### 3.3. Difference of Urinary Cortisol in Giant Pandas under Different Feeding Modes

**Figure 2:**
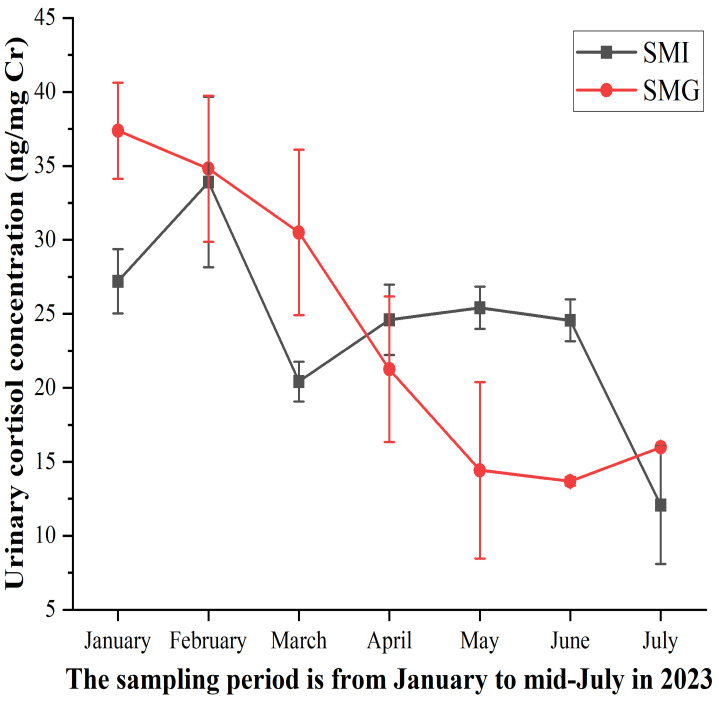
**Cortisol statistics of two groups of giant pandas.** The abscissa indicates the month, covering a span of seven months. The ordinate represents cortisol concentration, with values expressed as averages. n = 3 per group.

The urinary cortisol concentration of two groups of giant pandas was monitored from January to mid-July in 2023, with the results presented in Figure 2. The cortisol levels in the SMG group were slightly higher than those in the SMI group during January, February, March, and July, although this difference was not statistically significant. Similarly, the cortisol levels in the SMI group were slightly higher than those in the SMG group during April, May and June, with no statistically significant difference.

### 3.4. Difference of Fecal Microorganisms in Giant Pandas under Different Feeding Modes

**Figure 3:**
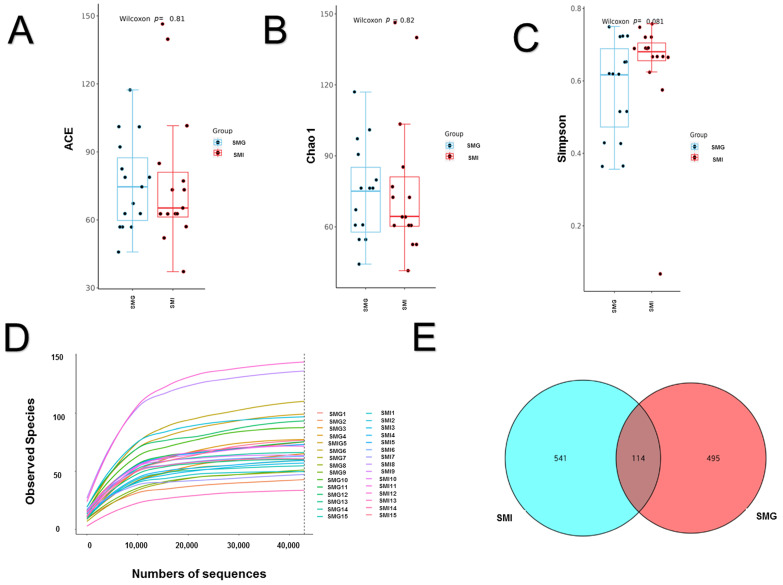
**Difference of fecal microorganisms between two groups of giant pandas.** (**A**) Represents ACE index. (**B**) Represents Chao1 index. (**C**) Represents Simpson index. (**D**) represents the dilution curve. (**E**) Represents Venn diagram. It showed that the pink area was the unique Amplicon Sequence Variants (ASVs) of a socially managed group (SMG). The green area was the unique ASVs of the solitarily managed individual group (SMI). The overlapping area in the middle is the ASVs shared by the two groups. n = 3 per group.

**Figure 4:**
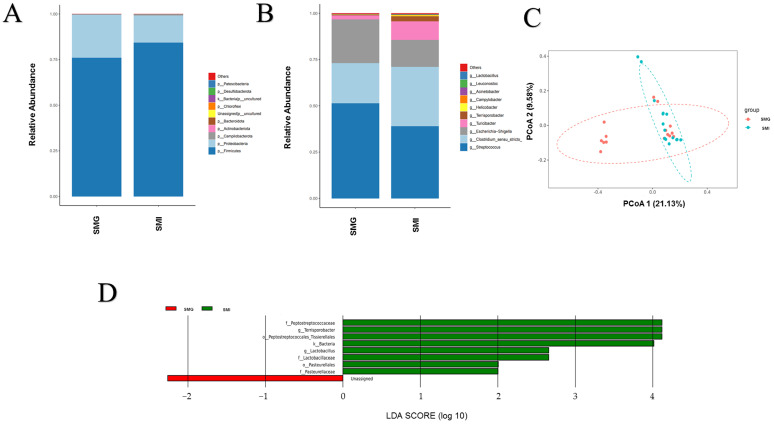
**16S rRNA: Microbial composition.** (**A**) Histogram of relative abundance of species at the level of each sample phylum. (**B**) Histogram of relative abundance of species at the level of each sample genus. Species with different metabolisms in different colors correspond to the legend on the right; the horizontal axis represents different samples or groups, and the vertical axis represents the relative abundance of different species. (**C**) PCoA analysis. The abscissa represents the first principal component, the ordinate represents the second principal component, and the percentage represents the contribution to the sample difference. (**D**) LDA Score obtained by LEfSe analysis. The red and green areas in the LDA value distribution histogram represent different groups; the red nodes in the branches represent the microbial groups that play an important role in the SMI group, and the green nodes represent the microbial groups that play an important role in the SMG group. Only the species with an LDA score greater than the set value (the default setting is 2) are shown in the figure, and the length of the histogram represents the size of the LDA value.

As depicted in Figure 3A–C, no statistically significant difference was observed in the ACE index, Chao1 index, and Simpson index between the SMG group and the SMI group of giant pandas. Of note, the rarefaction curve serves as a direct reflection of sequencing data rationality. Illustrated in Figure 3D, all dilution curves within our samples exhibit a tendency towards flatness, accurately portraying species and structural diversity within the jejunum microbial community. The β diversity of the samples was assessed using unweighted UniFrac index and non-metric multi-dimensional scaling (NMDS) analyses. The differences between the community structures of the groups were analyzed by the anosim analysis significance test (based on the Bray-Curtis algorithm). The results of the anosim analysis were R2 = 0.124952, *p* < 0.05. The principal coordinates analysis (PCoA) diagram illustrated distinct regions for the microbiota of SMG and SMI groups, except for individual samples (Figure 4C).

As depicted in Figure 3E, the fecal microbiota of giant pandas in the SMI group exhibited a total of 655 Amplicon Sequence Variants (ASVs), while the SMG group showed a total of 609 ASVs. Of these, there were 114 shared ASVs between the two groups, representing 9.91% of the overall ASVs. The SMI group displayed 541 unique ASVs, accounting for 47.04% of the total ASVs, whereas the SMG group presented 495 unique ASVs, constituting 43.04% of the total ASVs.

The fecal flora distribution (phyla and genus level) in the SMI and the SMG groups of giant pandas was depicted in Figure 4A,B. At the phylum level (Figure 4A), both groups exhibited a high abundance of Firmicutes and Proteobacteria, with no significant differences between the SMI and the SMG groups. At the genus level (Figure 4B), Streptococcus, Clostridium_sensu_stricto1, Escherichia-Shigella and Turicibacter were found to have higher abundance in both groups. Similarly, no significant differences were observed between the SMI and the SMG groups for these four bacterial groups.

Based on the above analysis of the community structure, we could determine that the microbial flora in the giant panda feces of the SMI group has undergone tremendous changes compared with the SMG group. We further used the linear discriminant analysis (LDA) to screen the differences. The differential strains were screened according to the criteria of LDA > 2 and *p* <0.05; the differential strains are screened. At the lower genus level, we observed that the abundance of genus *Terrisporobacter* and family Peptostreptococcaceae increased significantly in the SMI group (*p* < 0.01) (Figure 4D).

## 4. Discussion

### 4.1. Influence of Social Environment on Giant Panda Behavior

There is a growing recognition of ex situ conservation as a collection of techniques that can facilitate the integrated management of both wild and captive populations [28]. Nonhuman animals in captivity exhibit behaviors and physiological conditions that are distinct from those observed in the wild [29,30]. In the context of ex-situ conservation efforts for the highly solitary and endangered giant pandas, there is a need to consider its social behavior during the sub-adult stage as a means to optimize space and management costs. Researchers have observed substantial alterations in the behavior of long-term captive wild animals, a phenomenon frequently interpreted as an adaptation to their environment [31]. Meng et al. (2012) discovered that within artificially established captive elk populations, individuals display three conflicting behaviors: attacking, displacing, and threatening. However, male captive elk establish a stable social hierarchy through increased aggressive behavior to assert their dominant position, a phenomenon not observed in the wild [32]. Subsequently, we will explore the behavioral adaptation exhibited by the solitary species when in a “gregarious” state.

Foraging is essential for the survival and reproductive success of animals [33]. The giant panda primarily relies on bamboo as its main source of energy and nutrients. In order to obtain even a small amount of these essential components, the giant panda must consume a large quantity of bamboo, sometimes up to tens of kilograms per day [34]. In their natural habitat, wild giant pandas allocate a significant portion (55%) of their daily activities to foraging in order to meet their energy requirements [35]. In contrast, captive giant pandas are provided with regular feedings by caretakers and do not engage in independent foraging. As a result, resting and feeding behaviors have become predominant, accounting for over 70% of their daily activities. Our research yielded similar findings, as both the SMG and SMI groups of giant pandas predominantly exhibit resting and feeding behaviors. In order to meet their energy demands, giant pandas must consume a sufficient amount of food [35].

While bears are typically nocturnal or crepuscular, they may also be active during the day. Polar bears (Ursus maritimus) are primarily diurnal [19]. Given that our behavioral observations were conducted during the daytime, it is possible that this influenced our finding that giant pandas spend most of their time engaged in feeding and resting behaviors. Our study did not find any statistical difference in feeding behavior between the two groups of giant pandas, suggesting that housing conditions may not alter their food requirements despite no longer needing to forage for themselves. Stereotypical behavior is a prevalent abnormality observed in captive animals, often attributed to the conditions of captivity and their impact on animal welfare [36,37,38]. Mason and Latham (2004) conducted a statistical analysis that revealed a correlation between the manifestation of stereotyped behavior and a decline in animal welfare [39]. Several environmental factors, such as spatial constraints, the impact of feeding schedules, limitations on environmental control, and a lack of stimulation, have been identified in association with the development of stereotypic behaviors [24]. Our study revealed a significant decrease in stereotyped behavior among giant pandas living in social groups compared to solitary individuals, potentially attributed to the substitution of such behaviors with playful activities. Furthermore, we advocate for the promotion of foraging behavior in giant pandas as it may serve as a replacement for their inflexible behaviors.

It is widely accepted that the playing behavior of animals is associated with positive emotions [40]. Playing behavior can be categorized as social or non-social [41], with social play involving group interactions and non-social play encompassing sports and physical games [42]. While there is substantial research on playing behavior in certain carnivorous animal families such as canids, felids, and hyenas, systematic investigation into bear games remains limited [43]. Play is undeniably crucial for captive animals, and Kleiman (1994) suggests that providing captive males with the opportunities for regular dominance interactions may enhance reproductive behavior [44]. Snyder et al. (2003) evaluated the impact of early separation from mothers on the behavioral development of giant pandas, highlighting that a lack of social stimulation from the mother led to reduced playing behavior and increased periods of inactivity in offspring [45]. Play is considered an indicator of a positive state, typically occurring when all other needs are met and diminishing when threatened [46]. In our investigation, the SMG group of giant pandas exhibited a significantly greater duration of engagement in playful behavior compared to the SMI group. It is regrettable that I did not categorize and quantify interactive playing behavior separately. However, based on my observations, it is evident that social pandas dedicate a significant portion of their time to engaging in interactive play behavior. The findings suggest that sub-adult pandas in captivity may experience improved welfare when housed in social groups rather than solitary conditions. While direct evidence is currently lacking, it is hypothesized that this type of social environment could potentially have enduring effects on the behavior of giant pandas.

### 4.2. The Effects of Group Breeding on HRV, Cortisol, and Fecal Microbiota in Giant Pandas

Numerous clinical studies have confirmed the association between chronic psychological stress and dysfunction of the hypothalamic pituitary adrenal axis (HPAA). Cortisol, an important hormone regulating HPAA function, is found to be elevated in individuals experiencing psychological discomfort [47]. Furthermore, chronic stress has been shown to impact the sympathetic adrenal medullary system (SAS) through its regulation of HPAA function [48], resulting in the release of adrenaline [45] and influencing human HRV levels [49,50]. Currently, there is a lack of research on assessing the psychological status of wild animals, particularly in evaluating the function of the sympathetic nervous system using HRV. While some zoos monitor the ECG of large captive animals under anesthesia, it is important to note that different anesthetics can impact ECG readings [51]. In our study, we conducted non-anesthetic ECG monitoring for giant pandas for the first time and analyzed the HRV data. Our findings revealed that the SDANN was 56.541 ± 23 ms in the SMI group and 29.505 ± 12 ms in the SMG group, with no significant difference between these two groups. Given the current lack of HRV data for giant pandas, although our results may not establish a normal range, they do contribute to filling this gap and offer a new approach to ongoing monitoring of giant pandas’ cardiac function.

Cortisol is commonly known as the stress hormone due to its increased production in response to severe psychophysiological stress, making it a widely used indicator for assessing animal welfare [52,53]. While some researchers have suggested that cortisol serves as an indicator of stress, it is important to note that welfare cannot be solely determined by the absence of stress [54,55]. However, our research findings do not appear to support this perspective. We conducted a study on the urinary cortisol concentration of two groups of giant pandas from January to mid-July in 2023 and found that there was no significant difference between the two groups. In other words, solitary pandas exhibiting stereotypical behavior (a negative indicator of well-being) demonstrated similar cortisol levels to those of group-living pandas. This suggests that the manifestation of stereotyped behavior in solitary giant pandas does not appear to be indicative of stress, as it does not elevate adrenal cortex activity. Instead, it may represent a behavioral adaptation to short-term solitary living due to the lack of opportunities for social interaction with peers, leading them to engage in alternative behaviors such as stereotypy to alleviate boredom [56].

Additionally, we analyzed the fecal microorganisms of both groups using high-throughput sequencing targeting 16S rDNA, aiming to explore the impact of sub-adult social and solitary environments on giant panda intestinal flora. Our results indicated that the group housing strategy markedly elevated the β diversity of gut microbiota in sub-adult giant pandas. Β diversity of the gut microbiota is likely very important to animal health [57]. Microbial diversity is actually a relatively stable state. In fact, the decrease in microbial diversity is a state of an unhealthy intestine, which is mostly caused by drastic changes in diet patterns, microbial infections and widespread use of antibiotics [23,58,59]. The advantages of communal living encompass heightened prospects for food acquisition, stress alleviation, lowered predation risk, and enhanced individual as well as collective physical fitness [60]. The symbiotic relationship between the host and gut microbiome has undergone co-evolution [61]. An organism’s lifestyle may exert an influence on its gut microbiome. Subsequent investigations have revealed that an animal’s group-living behavior can result in augmented richness and diversity within its gut microbiome, promoting the proliferation of beneficial bacteria while diminishing harmful strains [62]. Similarly, our findings also demonstrate a significantly higher abundance of pathogenic bacteria genus *Terrisporobacter* and family Peptostreptococcaceae in the intestines of solitary-housed sub-adult giant pandas compared to those housed in groups, suggesting that group housing may be more conducive for captive sub-adult giant pandas.

## 5. Conclusions

In conclusion, the group-rearing model during the sub-adult period resulted in a significant reduction in stereotypic behavior and a notable increase in playing behavior, as well as an enhancement of β diversity within the gut microbiota of captive giant pandas. However, no significant impact was observed on the HRV indicators and cortisol levels related to stress in captive sub-adult giant pandas. In conclusion, within the present captive setting, the group-rearing approach during the sub-adult stage proved less distressing for adult captive giant pandas.

## Figures and Tables

**Table 1:** The information of giant pandas.

Group	Name	Studbook	Birth	Sex	Sir	Dam	Time of Capture of Behavioral Data
SMI	Qi Hang	1132	2018	Male	Xiong Bing	Qing He	2023.7.24–2023.7.29 (24 h)
SMI	Qi Cheng	1131	2018	Female	Xiong Bing	Qing He	2023.8.15–2023.8.21 (24 h)
SMI	Fu Wa	948	2015	Female	Qiao Qiao	Ke Lin	2023.9.12–2023.9.18 (24 h)
SMG	Guo Guo	1252	2020	Female	Xiong Bing	Qing He	2023.7.24–2023.7.29 (24 h)
SMG	Qiang Qiang	1251	2020	Male	Xiong Bing	Qing He	2023.8.15–2023.8.21 (24 h)
SMG	Fu Shuang	1250	2020	Female	Gong Zai	Fu Lu	2023.9.12–2023.9.18 (24 h)

Note: SMI represents the solitarily managed individuals; SMG represents the socially managed groups.

**Table 2:** The definitions of behaviors to observe in the directly captive sub-adult giant pandas.

Behavior Category	Behavior	Definition
feeding behavior	take food drink water	Eat bamboo, bamboo shoots, steamed bread, etc., drink water.
resting behavior	rest	Maintain a stationary state in various postures, with or without closed eyes.
playing behavior	play with objects	Play with objects such as bamboo sticks, twigs, toys or water.
	self-play	Including backward rolling and swinging limbs, forward rolling, inverted spinning around objects, and climbing. Climbing walls and railings, etc.
	interactive play	Two or more giant pandas play with each other.
stereotyped behavior	bite the rail	Repeatedly gnawing on the railing of the enclosure, rubbing against it, or even sucking on it.
	step by step	Constantly moving back and forth in the enclosure.
	turn around	A highly specialized pacing behavior that accurately follows the same route every time (circle).

Note: This table referred to Refs. [8,23,24,25].

**Table 3:** Heart rate variability of two groups of giant pandas.

Index	SMI	SMG	*p*-Value
SDANN	56.541 ± 23	29.505 ± 12	>0.05

**Note:** SDANN refers to the mean standard deviation of the R-R interval, measured in milliseconds, and is an indicator of HRV. SMI represents separate feeding groups; SMG represents the mixed (social) feeding group. A *p*-value > 0.05 is considered insignificant between the two groups.

## Data Availability

The datasets presented in this study can be found in online repositories. The names of the repository/repositories and accession number(s) can be found below: NCBI—PRJNA1155169.

## Abbreviations

HRV—heart rate variability; SDNN—standard deviation of normal-to-normal intervals; ECG—Electrocardiogram; HPAA—hypothalamic pituitary adrenal axis.
